# Intrathoracic fire during preparation of the left internal thoracic artery for coronary artery bypass grafting

**DOI:** 10.1186/1749-8090-5-10

**Published:** 2010-03-10

**Authors:** Martin Friedrich, Theodor Tirilomis, Jan D Schmitto, Aron F Popov, Suyog A Mokashi, Marc Hinterthaner, Gunnar G Hanekop, Paul Zwaka, Friedrich A Schoendube

**Affiliations:** 1 Department of Thoracic and Cardiovascular Surgery, University of Göttingen, Göttingen, Germany; 2 Division of Cardiac Surgery, Brigham and Women's Hospital, Harvard Medical School, Boston, MA, USA; 3 Department of Anesthesiology and Intensive Care Medicine, University of Göttingen, Göttingen, Germany; 4 Department of Radiology, University of Göttingen, Göttingen, Germany

## Abstract

A surgical fire is a serious complication not previously described in the literature with regard to the thoracic cavity. We report a case in which an intrathoracic fire developed following an air leak combined with high pressure oxygen ventilation in a patient with severe chronic obstructive pulmonary disease. The patient presented to our institution with diffuse coronary artery disease and angina pectoris. He was treated with coronary artery bypass graft surgery, including left internal thoracic artery harvesting. Additionally to this rare presentation of an intrathoracic fire, a brief review of surgical fires is included to this paper.

## Introduction

While surgical fires are rare, they can potentially result in significant morbidity and mortality [1-3]. A comprehensive literature search revealed only a limited number of intraoperative fires. The vast majority of cases involved endotracheal ventilation and the use of electrocautery or laser. Surgical fires are created with an igniting source (an oxidizer and fuel). Igniting agents include electrocautery or laser, which combine with either oxygen or nitrous oxide as the oxidizer. The fuel for the fire may be the surgical drapes, prepping agents, or even human tissue itself [2,4,5]. We present an interesting case of an intrathoracic fire in a patient undergoing LIMA harvesting during CABG surgery.

## Case Report

A 57-year-old male was presented to our institution with angina pectoris. The patient was significant for chronic obstructive pulmonary disease (COPD), for which he was receiving maximal medical therapy - beclometasone, formoterole, and salbutamole. Results of the incentive spirometry summarized in Table 1. Cardiac catheterization established the presence of severely calcified coronary disease with multiple high grade stenoses of the left anterior descending and circumflex artery, along with middle grade stenoses of the right coronary artery. The patient was taken to the operating room for CABG surgery.

**Table 1:** Spirometry before surgery.

	Expected Value	Measured Value	Expected/Measured
**Vital Capacity**	4.03 L	2,38 L	59%

**FVCex**	3.78 L	2.62 L	68%

**FEV1**	3.01 L	0,78 L	26%

**FEV1/VCmax**	75%	30%	39%

**RV**	2.51 L	6.26 L	249%

**Resistance**	< 0.30 kPa/L	0.73 kPa/L	245%

VC: vital capacit; FEV1: forced exspired volume in 1 second; RV: residual volume

**Figure 1:**
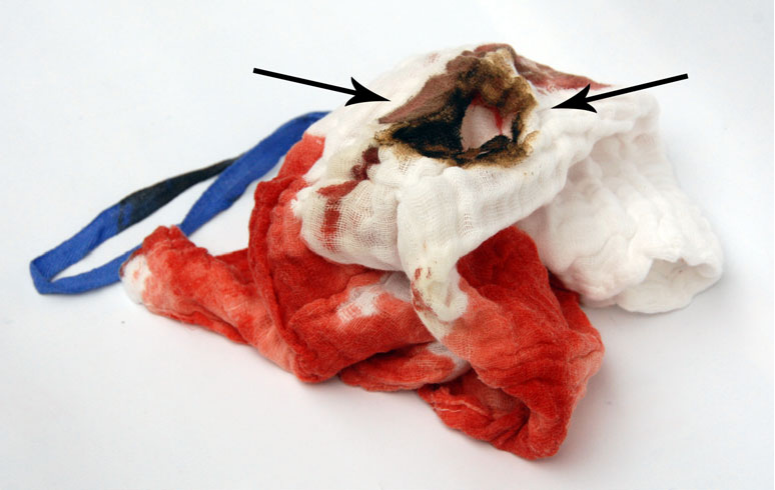
**Marks of fire on the used surgical sponge after burning in the chest (indicated by arrows)**.

After the patient was successfully intubated, general anesthesia was maintained with sufentanil, propofol, and sevoflurane. After median sternotomy, the oxygen saturation dropped to 82%. At this point, a pneumothorax was noted, both pleura were immediately opened and the FiO_2 _was increased from 0.7 to 1.0 (high pressure ventilation). On examination, the lungs contained several small blebs with a large, intact bulla in the basal segments of the left lower lobe. We were not able to detect a laceration on the lungs, however, the respirator (closed system; Zeus Aryl-0021, Dräger, Luebeck, Germany) signaled an air leakage of 1.2 L/min. The ventilator was modified to decrease the air leakage to 1.0 L/min (FiO_2 _1.0). We then proceeded with the surgery and began harvesting the LITA with the pedicle technique using electrocautery (Erbe VIO 300D, Medizintechnik GmbH, Tuebingen, Germany). A gauze sponge was used to compress the prominent upper lobe to facilitate LIMA preparation. Instantaneously, the gauze sponge began burning and a small flame developed in the thoracic cavity. The sponge was quickly removed from the chest (Fig. 1). Even after sponge removal, the preparation area of the LITA continued to burn. We extinguished the fire manually with gauze soaked in cold saline. We then thoroughly inspected the LITA and left upper lobe - neither was damaged and the air leakage did not increase. The remainder of the operation was uneventful, and the patient left the operating room in stable cardiopulmonary condition on low dose catecholamines. Three hours later he was extubated in the intensive care unit.

**Figure 2:**
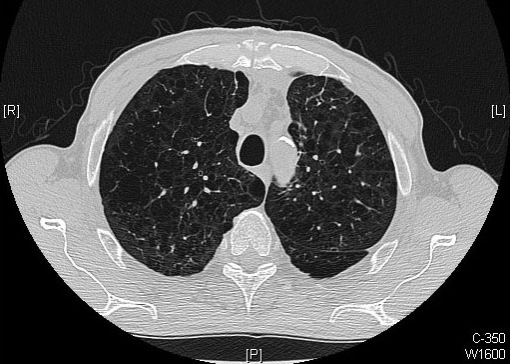
**Postoperative computer tomography scan of the chest**.

On the first postoperative day the patient was transferred from the intensive care unit to the normal ward. Two days later, the air leakage increased, however he denied any symptoms of dyspnea. However, given the patient's severe emphysema, we decided to perform an exploratory thoracotomy to identify the persistent air leakage. Intraoperative small lesions on the posterior part of the left upper lobe were noted and oversewn, and securely sealed with human fibrinogen. Three days later a talcum pleurodesis was performed. Over the next week, the air leak progressively decreased and the chest drainage was removed. Thoracic computer tomography scan demonstrated multiple bilateral lung bullae without evidence of pneumothorax (Fig. 2). The patient was discharged on postoperative day 21. At four-weeks follow up, he remained well.

## Discussion

The consequences of a surgical fire can be fatal [6-8]. Surgical fires can potentially happen whenever an invasive procedure is performed [9,10]. There are very few reported cases of fires caused by a leaking oxygen connection in operating room [1,2]. In other cases surgical fires resulted next to the airways [6,11]. Under special conditions in the surgical environment the combination of: 1) oxygen, 2) an ignition source and 3) inflammable material - three essential elements - may result in a surgical fire [1,2,7]. In critical situations oxygen-rich ventilation is often needed. The use of 100% oxygen during episodes of hypoxemia is a cornerstone of anesthetic management. Additionally, in cases of open airways (e.g., tracheal, carinal, and bronchial resections) continuous positive airway pressure is often applied with preferred air-mixture of 100% oxygen to improve oxygenation [11]. In such cases we must understand that high oxygen concentrations in closed spaces could increase a risk of fire [8,9,12]. It is important to note that under normal circumstances, modern inhalation anesthetics are not ignitable [13].

In the present case sevoflurane was used. Electrocautery or laser may have led increased the temperature near or above the ignition temperature [14,15]. Aly et al. [7] reported a fire caused by electrocautery when it entered in to trachea, probably due to the high pressure stream of oxygen passing over the hot electrocautery tip and hot charred tissue. Casey and collaborators [6] reported an intratracheal fire ignited by Nd-YAG laser during treatment of tracheal stenosis. In our case, a lightbow initiated by electrocautery resulted in the fire of a surgical gauze sponge and the space around it. An alternative to electrocautery may be ultrasonic scalpels [16,17] - the harmonic scalpel which contains a transducer system using piezoelectric crystals. Markovicz and colleagues [15] found that bipolar electrosurgery produced ten times more tissue damage than ultrasonic energy. Their study also showed that the harmonic scalpel produced less than 1°C temperature increase in the area of the LITA.

We did not expect that harvesting the LITA would induce a fire in a surgical sponge, given the thorax was open. The case demonstrates that pulmonary leakage may result in a high oxygen saturation of the pleural space, which may result in combination with electrocautery in burning of surgical materials/tissue. To avoid another occurrence in a similar case during harvesting of the LITA with an air leak we recommend the use of a wet surgical sponge for compression of the left lung.

## Conclusion

In conclusion, the combination of the three components: fuel, oxygen, and ignition can induce a fire in the surgical field. This case points out the importance of persistent vigilance and quick reaction to prevent injury in the operating theater.

## Competing interests

The authors declare that they have no competing interests.

## Authors' contributions

MF performed the first step of operations, directed postoperative Care, wrote and revised manuscript, TT did data interpretation and wrote and revised manuscript, JDS did data interpretation and revised manuscript, AFP assisted with the operation, participated in postoperative patient care and collected data and revised manuscript, SAM did data interpretation and revised manuscript, MH performed the second step of operation and revised manuscript, GGH supervised intraoperative and postoperative anesthesia care and revised manuscript, PZ did interpretation of X-Rays and revised manuscript, FAS participated in its coordination and revised manuscript. All authors read and approved the final manuscript.

## Consent statement

Written informed consent was obtained from the patient for publication of this case report and accompanying images. A copy of the written consent is available for review by the Editor-in-Chief of this journal.
